# Immune-endocrine biomarkers associated with mental health: a 9-year longitudinal investigation from the Hertfordshire Ageing Study

**DOI:** 10.1192/j.eurpsy.2022.327

**Published:** 2022-09-01

**Authors:** R. Hou

**Affiliations:** University of Southampton, Department Of Psychiatry, Southampton, United Kingdom

## Abstract

**Introduction:**

Ageing is accompanied by the development of low-grade systemic inflammation which may promote changes in the neural systems predisposing to geriatric depression via the hypothalamic-pituitary-adrenal (HPA) axis.

**Objectives:**

The aim of this study was to investigate the longitudinal associations between baseline values and conditional changes in immune-endocrine biomarkers and mental health status in a population-based cohort of older adults.

**Methods:**

Data from 347 subjects(200 men, 147 women) who participated in the Hertfordshire Ageing Study at baseline(mean age 67.3 years) and at 9-year follow-up were analysed. Serum samples for analysis of inflammatory and endocrinological measures were collected at baseline and follow-up. At follow-up, depression (Hospital Anxiety and Depression Scale) and mental health(Short Form-36 questionnaire) were assessed. Baseline values and changes in biomarkers in relation to risk of high depression scores and low mental health scores were examined using logistic regression.

**Results:**

Lower baseline cortisol was related to greater risk of high depression scores; higher baseline cortisol: Dehydroepiandrosterone Sulphate ratio(men only) and higher baseline CRP(women only) were related to greater risk of poor mental health scores. In addition, greater decline in cortisol was related to increased risk of high depression scores among men. These relationships were robust(p<0.05) after controlling for sex, age, BMI, smoking, alcohol consumption and number of systems medicated.

**Conclusions:**

This study provides further evidence of the role of the HPA and inflammation in older adults with poor mental health. In addition, the findings highlight sex differences where increased inflammation in women and declines in cortisol in men was linked to poorer mental health.

**Disclosure:**

No significant relationships.

